# Persian version of the Barkin Index of Maternal Functioning (BIMF): a cross-cultural adaptation and psychometric evaluation

**DOI:** 10.1186/s12884-021-03556-4

**Published:** 2021-01-25

**Authors:** Mehri Ansariniaki, Minoor Lamyian, Fazlollah Ahmadi, Abbas Rahimi Foroushani, Carolann L. Curry, Jennifer L. Barkin

**Affiliations:** 1 Department of Midwifery and Reproductive Health, Faculty of Medical Sciences, Tarbiat Modares University 14115-111, Tehran, Iran; 2 Department of Nursing, Faculty of Medical Sciences, Tarbiat Modares University 14115-111, Tehran, Iran; 3 Department of Epidemiology and Biostatistics, School of Public Health, Tehran University of Medical Sciences 6446, Tehran, Iran; 4 Mercer University School of Medicine, Mercer University, 1550 College Street, Macon, GA 31207 USA

**Keywords:** Psychometrics, Barkin-index of maternal functioning, Maternal health, Postpartum functioning, Postpartum mental health

## Abstract

**Background:**

Postpartum maternal functioning has the potential to affect the quality of interaction between mother and child. A proper assessment of maternal functioning requires a comprehensive and accurate tool. The objective of this study was to prepare a Persian version of the Barkin Index of Maternal Functioning (BIMF) and evaluate its psychometric properties in order to determine its applicability in Iranian mothers.

**Methods:**

The BIMF was translated into Persian and then culturally adapted for Iranian women. After evaluating face and content validity, to perform factor analysis, a cross-sectional study was conducted using the Persian version of BIMF. The data was collected from two unique groups of 250 mothers (in all 500 mothers) who had infants 2 to 12-months old and who were selected using a two-stage cluster sampling method. Factor analysis, Pearson’s correlation, intra-class correlation coefficients (ICC), composite reliability (CR) and Cronbach’s alpha were employed in order to evaluate structural validity and reliability.

**Results:**

Exploratory factor analysis resulted in a five-factor structure consisting of 20 items. Subsequently, confirmatory factor analysis (X ^2^/ df = 1.61, RMSEA = 0.050, GFI = 0.91, CFI = 0.91) confirmed that the Persian version had satisfactory goodness of fit. Reliability and internal consistency were confirmed with a CR of 0.77, an ICC of 0.87 and a Cronbach’s alpha of 0.81.

**Conclusions:**

The findings indicated that the Persian version of the BIMF is a valid and reliable instrument for assessing maternal functioning among Iranian mothers.

**Supplementary Information:**

The online version contains supplementary material available at 10.1186/s12884-021-03556-4.

## Background

Maternal functioning after childbirth refers to the application of certain skills that mothers acquire in order to master their role as the primary caregiver of the child, ensure their own health and play a lead role in the management of a household [1]. Barkin et al. identified the key functional domains of a mother during the postnatal period as: self-care, infant care, mother-child interaction, psychological wellbeing of mother, social support, management, and adjustment [2–4]. While the deleterious effects of postpartum depression on offspring are well established [5], the impact of impaired postpartum functioning on offspring and the family unit as a whole must be explored. Though not the topic of this article, it is necessary to elucidate the effect of impaired maternal functioning on long-term growth and development in affected children [6]. Measurement of postpartum functioning is also important as an alternative (when appropriate) or as a complement to Perinatal Mood and Anxiety Disorders (PMADs) evaluation and treatment. Often, when women present for treatment, they express interest in improved daily functioning rather than achievement of a specific score on a depression assessment [2]. In order to accurately capture maternal functional status for both clinical purposes and academic research, a valid, brief, patient-centered tool is required [7].

Fawcett et al. (1988) initially conceptualized and defined postpartum maternal functioning by developing the first proprietary tool to capture the construct namely the Inventory Functional Status After Childbirth (IFSAC), which consists of 36 items and five factors [8, 9]. Despite the multidimensionality and relatively widespread use [9–14], the IFSAC has considerable deficits. The primary detractor is the scoring algorithm, which inherently penalizes women who have not returned to all of their pre-birth activities. This premise for characterizing women’s functional levels is flawed as maternal reprioritization is often necessary and healthy. This is also a sign that the woman is aware that adjustment is required to accommodate a new life [2, 3]. Additionally, all relevant functional domains are not represented within the IFSAC. In fact, maternal psychological wellbeing is largely neglected as the IFSAC appears to be more a task-based instrument [15–17]. The 36-item format has also proven cumbersome in the context of clinical trials where participants are completing multiple assessments at once [Barkin, J.L. Personal communication].

In 2010, Barkin et al. published the first of several foundational articles for the development of the Barkin Index of Maternal Functioning (BIMF), a 20-item self-report measure intended to capture maternal functional status in the first 12 months postpartum [2, 3]. A grass root, patient-centered, approach was used to inform the item content for the BIMF. Specifically, a focus group study (*n* = 31) was conducted in order to define the functional spectrum from new mothers’ point of view. In the study, women were asked to describe the circumstances surrounding low, moderate and high functioning days [3]. The prominent, recurring focus group themes were transformed into questions for future respondents to answer with respect to their experience over the prior 2 weeks [2].

The BIMF has been validated in various subgroups of the population and internationally [18–21], is simply worded and easily administered [22], and has been translated into over 20 languages [23]. However, to our knowledge at the beginning of the study, only the Turkish version of the BIMF has been culturally adapted (subsequent to the initial translation) and validated in the corresponding population of women [7]. Cultural adaptions are essential to ensure that the translated version of the index resonates with the population of interest; the process of cultural adaption also mitigates the risk of misinterpretation on the part of the respondent, as even seemingly slight differences in the usage/meaning of words from one culture to another may affect how the participant responds to the questions [24]. For example, item 2 on the English version of the BIMF states, “I feel rested” and requires the participant to indicate to what degree they agree with this statement. However, in some cultures, “rested” means “at peace” which was not the developer’s intended meaning. Though cultural adaptations may only entail slight wording changes with most of the meaning left intact, it is prudent to also re-evaluate reliability, validity and factor structure in the modified version.

The importance and role of postpartum mothers’ functioning status on optimal mother-child interaction has been emphasized in many studies [25, 26]. Given the significance of the construct, the aim of this investigation was multi-fold and includes: 1) Preparation and cross-cultural adaptation of the Persian version of the BIMF and an examination of 2) face validity, 3) content validity, 4) construct validity, and 5) factor structure of the adapted measure in two unique groups of 250 Iranian mothers.

## Methods

### Design

This study was conducted in two phases. In the first phase, the BIMF was translated into the Persian language and cultural adaptation was performed. During the second phase, psychometric properties of the Persian version of BIMF were assessed and validated in Iranian mothers.

### The questionnaire

Original questionnaire***,*** **t**he Barkin Index of Maternal Functioning (BIMF) is a self-reported questionnaire designed to measure maternal functioning status. It was developed by Barkin et al. and consists of 20 items and 7 domains that were developed based on a holistic, patient-centered approach with Cronbach’s alpha of 0.87 [8, 27]. Each item is rated on a 7-point Likert scale ranging from 0 = “strongly disagree” to 6 = “strongly agree”. The total score ranges from 0 to 120. Higher levels of functioning are associated with higher total scores with 120 representing optimal functioning [2].

### Translation and cultural adaptation processes

The original English version of the BIMF was prepared and translated into Persian after obtaining permission from the developer (Jennifer L. Barkin, PhD). The basis of this phase of the study was the World Health Organization (WHO)‘s Process of Translation and Adaptation of Instruments [28]. In addition, the International Quality of Life Assessment (IQOLA) project [29] has also been used. In the first stage, two fluent translators in both languages (one of who had a medical background), translated the original questionnaire with a focus on conceptual translation from English into Persian using forward translation [28]. In the second stage, the research team and translators identified difficult items and substituted with correct words where appropriate. At the same stage, to assess the quality of the translation, two other translators evaluate the translation for language and conceptual equivalence. The revised translation was again examined by the research team. An evaluation of the agreement was carried out in order to assess the quality and difficulty of the primary Persian version. Finally, the Persian version of the BIMF with desirable quality was obtained. In the third stage, two new translators translated the secondary Persian version into English using backward translation. These translators were not acquainted with the original version of the questionnaire, but were fluent in both languages. The revised version of the questionnaire was reviewed by the research team and two English language teachers. Then a satisfactory English translation was sent to the BIMF developer for confirmation. After making necessary modifications, the final English version was returned into Persian (Additional file 1).

### Psychometric properties of the Persian version of the BIMF

(i) Content validity: Using the qualitative method, ten specialty experts in gynecology, midwifery, reproductive health and maternal and child health were invited to review and provide suggested edits for the questionnaire [30]. The edited questionnaire was then sent back to the specialty experts for approval. Quantitative content validity was assessed using the same ten experts based on specific forms of the content validity ratio (CVR) and the Content Validity Index (CVI). According to the Lawches᾿ table, a CVR score above 0.62 (for 10 experts) indicated a necessary and important questionnaire item [31]. The CVI was also used to determine the relevancy, simplicity, and clarity of items using a 4-point Likert scale rated by the ten experts. A CVI score above 0.79 was considered to be appropriate [30, 31].
(ii) Face Validity: In order to examine qualitative face validity, the principal investigator conducted in person interviews with 20 target group participants. The items were edited based on the recommendations of this group until no new recommendations were suggested. The purpose of this process was to simplify the item wording [30]. In order to examine quantitative face validity, a 5-point Likert scale was used for each of the 20 questionnaire statements, with “strongly important” scored as 5, and “not at all important” scored as 1. The impact scores for each of the items were calculated and values more than 1.5 were considered to be appropriate [32].
(iii) Concurrent validity: In order to examine concurrent validity, we compared the BIMF to another instrument that measures maternal functioning. For this purpose, the Inventory Functional Status After Childbirth (IFSAC) was used as a comparator. The IFSAC is a self-report questionnaire containing 36 items intended to tap postpartum functional status and the first calculated Cronbach’s alpha was reported as 0.76 [8]. The IFSAC assesses the ability or readiness of mothers to assume infant care responsibilities and to resume self-care responsibilities, household, occupational, social and community activities [33]. Items on the IFSAC are rated on a 4-point scale. In the case of the self-care and occupational activities subscales, 1 corresponds with “never” and 4 corresponds with “all of the time”. For all other subscales, 1 reflects “not at all” and 4 reflects “fully” [34]. Participants completed the two questionnaires simultaneously and the Spearman correlation analysis was performed. The least acceptable correlation was considered to be 0.7 [35].
(iv) Structural validity: Initially, the exploratory factor analysis (EFA) was conducted using a sample group (*n* = 250). In order to confirm the factor structure obtained, CFA was performed using another sample group (*n* = 250).

### Data collection

Data collection instruments consisted of a sociodemographic and reproductive characteristics questionnaire and the BIMF, which were completed by the participants from the second postpartum month up to the twelfth. Prior to data collection (from November 2018 to March 2019) the researchers explained the aims of the study to mothers referring to health centers and obtained written consent. Then, after explaining how to respond to the questionnaire, the questionnaire was provided to the participants.

### Study participants and sampling method

Participants were selected from a population of mothers referred to 30 health centers in Tehran (the capital of Iran) and Semnan (center of Semnan province in the central part of Iran). For sample selection, a two-stage random cluster sampling method was used. In the first stage, health centers were divided into three segments covering Tehran, Iran and Semnan Universities of Medical Sciences. Subsequently, 10 centers were randomly selected in each segment. In the second stage, samples were selected from each center proportional to the population attending that center. Inclusion criteria included women: 1) over 18 years of age, 2) literate in Persian, 3) Iranian citizens, 4) living in households in Tehran or Semnan, 5) with children between the ages of 2 to 12 months (regardless of number of deliveries and type of delivery), 6) who have given birth to a singleton and term (37–42 gestation weeks) infant, 7) who have no severe mental and physical illness (as declared by the participant), and 8) who were willing to participate in the study. The sample size was estimated based on the number of items in the questionnaire. Since the BIMF has 20 items, at least 3 to 15 participants are recommended for each item [36]. Therefore, 250 individuals were considered adequate for each exploratory and confirmatory factor analysis.

### Data analysis

Descriptive statistics were used to describe sociodemographic and reproductive characteristics and maternal functioning. Inferential statistics were used to determine the validity and reliability of the Persian version of BIMF. The statistical software used for data analysis was SPSS 25 (IBM, Armonk, NY) and AMOS 22.0 (IBM, Armonk, NY, USA). The EFA, based on the results of a sample group (*n* = 250), was performed using principal component analysis (PCA) and varimax rotation. Kaiser-Meyer-Oklin (KMO) values greater than 0.6 were considered as the sample size adequacy criterion. The Bartlett sphericity test with a confidence level of 95% or higher was considered the criterion of suitability of the data for performing EFA [37]. Items with factors loadings ≥0.40 were considered acceptable to belong to a given factor. To confirm the EFA results, based on the results of another sample group (*n* = 250), the CFA was performed using the Maximum Likelihood method and multiple goodness of fit indices. The indices used in this study and their acceptable values to confirm the goodness fit of model were as follows: x^2^ / df ratio < 2, residual mean square error approximate (RMSEA) <  0.06, goodness of fit (GFI) > 0.90 and comparative goodness of fit index (CFI) > 0.90 [38]. The reliability of the Persian version of BIMF was evaluated in terms of internal consistency and stability. Internal consistency using the Cronbach’s alpha coefficient and composite reliability (CR) was calculated. The stability of the questionnaire was determined using the intraclass correlation coefficient (ICC) via test–retest reliability method. Test-retest reliability was evaluated among 30 mothers who completed the questionnaire twice within a period of 2 weeks. Values of Cronbach’s alpha, ICC and CR equal or greater than 0.70 were considered acceptable [39, 40].

## Results

### Description and characteristics of the participants

In order to perform EFA, two hundred and fifty eligible mothers completed the Persian version of the BIMF questionnaire and there were no missing data. The mean age of participants was 31.7 ± 5.7 years (Table 1). Most participants were stay-at-home mothers (76.8%) and had a high school diploma or higher (61.6%). The demographic characteristics of participants are displayed in Table 1.

**Table 1:** Demographic Characteristics of Study Population (*n* =)

	Mean (SD)	No. (%)
**Mother’s age (years)**	31.70 (5.70)	250(100)
**Infant’s age (months)**	6.71 (3.43)	250(100)
**Number of children**	1.57 (0.69)	250(100)
**Education level**		
Primary		26(10.40)
Secondary		224(89.6)
**Employment status**		
Employed		58(23.20)
Housewife		192(76.80)
**Unwanted pregnancy**		26(10.40)
**Unwanted sex of baby**		44(17.60)

### Maternal functioning

The mean score of Persian version of BIMF (*n* = 250) was 93.09 ± 13.30. The highest mean score was for the Infant Care factor (88.07 ± 16.55) while the lowest mean score was for the Psychological Wellbeing factor (55.27 ± 28.71)) (Table 2).

**Table 2:** The mean scores of the Persian version of BIMF and its factors (*n* = 250)

Persian version of BIMF	Mean^a^ (SD^b^)	Mean (SD)	Possible range	Obtained range
Satisfaction with Maternal Competence	84.38 (11.32)	45.57(6.12)	0–54	25–54
Self-care	75.56 (17.86)	18.14(4.29)	0–24	4–24
Infant Care	88.07 (16.55)	10.57(1.99)	0–12	1–12
Social Support	67.27 (21.93)	12.11(3.95)	0–18	0–18
Maternal Psychological Wellbeing	55.27 (28.71)	6.70(3.45)	0–12	0–12
**Total**	**77 (11.21)**	**93.09 (13.30)**	**0–120**	**47–119**

^a^Response scale range is 0–100

^b^Standard Deviation

### Content validity

As a result of our consultation with 10 experts, minor modifications were made to item 8 and item 15. Specifically, item 8, “I am getting enough adult interaction” was changed to, “I have enough communication with adults”. Item 15, “My baby and I are getting into a routine” was changed to, “I and my baby have a specific daily schedule”. It is important to note that no item was deleted from the modified questionnaire. The results of CVR indicated that all items had higher CVR scores (from 0.8 to 1.0) than the Lawshe table criterion (0.62 for 10 experts). All items were also identified as essential in assessing maternal functioning status and had acceptable statistical significance (*p* <  0.05). In the CVI section, the results indicated that all items had a CVI score higher than 0.79 (between 0.87 and 1). This indicates that the Persian version of the BIMF is suitable for measuring maternal functioning status.

### Face validity

In terms of qualitative face validity, several minor changes to the wording of Items 7, 11, and 20 were suggested by the 20 participants in the target group and prompted modifications. Specifically, the phrase “a little time” in Item 11, “I take a little time each week to do something for myself”, and the phrase “a new mother” in Item 20, “I am satisfied with the job I am doing as a new mother”, were misinterpreted by the participants. Because of this, Item 11 was changed to “During the week, I also take time to do my personal work”; Item 20 was changed to, “I am satisfied with my performance as someone who has recently had a baby”. BIMF items with impact scores greater than 1.5 were identified as important to the mothers in the target group in an analysis of quantitative face validity.

### Concurrent validity

An examination of the correlation between the Persian version of the BIMF and the IFSAC, yielded a coefficient of 0.77. This indicated a positive, and direct relationship as one would expect.

### Exploratory factor analysis

**Fig. 1:**
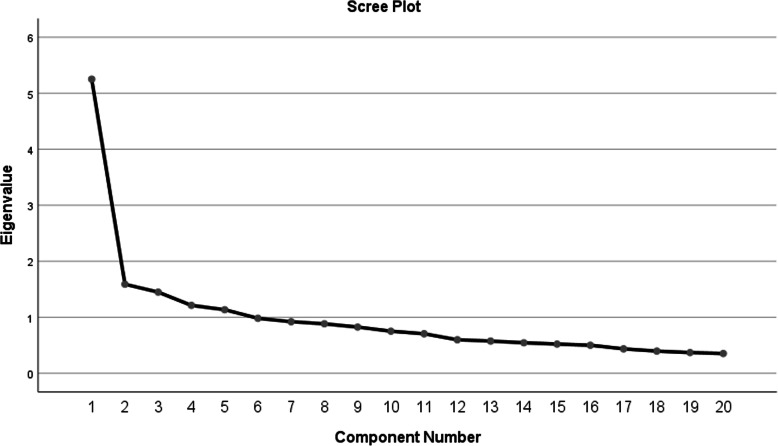
Scree plot for factor components of the Persian version of BIMF

The EFA results showed that the KMO value was 0.84, well above the recommended value of 0.6, indicating sampling adequacy. Bartlett’s test of sphericity, which indicates the suitability of the EFA application of the analysis to the data, was significant (χ 2 = 1,613,219; df = 190; *p* < 0.001). The EFA, using PCA, revealed five components with Eigenvalues greater than 1 and over half (53.19%) of the total variance of the BIMF was explained by these five factors (Table 3). The number of factors was also confirmed by the scree plot (Fig. 1). All questions had a minimum factor loading (equal to 0.4 based on a sample size of 250) [38]. After identifying and reviewing the items related to each factor, the five factors were titled: Satisfaction with Maternal Competence, Self-care, Infant Care, Social Support and Psychological Wellbeing (Table 3).

**Table 3:** Factor loadings for exploratory factor analysis with varimax rotation of the Persian version of BIMF

Items	Factor1	Factor 2	Factor 3	Factor 4	Factor 5
1. I am a good mother.	**.663**	.084	−.075	.118	.157
2. I feel relieved (rested).	**.631**	.300	−.043	.187	.132
3. I am satisfied with the way (whether by bottle, through breastfeeding, or both) that I have chosen to feed my baby.	.125	−.051	**.713**	.051	.151
4. My child and I understand each other.	**.552**	.112	.239	.091	−.083
5. I can calmly enjoy the time that I spend with my baby.	**.585**	.241	.239	−.074	−.015
6. If I need rest, I can leave my baby’s care without worry to people that I have in my life.	.194	.070	.059	**.736**	.021
7. I can comfortably leave my baby’s care to my trusted friends or relatives (This can include the father of the baby or spouse).	−.022	.155	.099	**.784**	−.095
8. I have enough communication with adult	.278	**.534**	−.060	.309	.052
9. I receive enough encouragement from others	.357	.284	.140	**.408**	.092
10. I believe in my inner sense (instincts) while taking care of my child.	**.627**	−.162	.225	.096	.028
11. In week, I dedicate a time to doing my personal work too.	.115	**.817**	−.017	.187	.048
12 I can fulfill my baby’s physical needs (such as nutrition, diaper changing, and bringing to the doctor).	.079	.162	.**693**	.115	−.059
13. I can fulfill my physical needs (such as showering, nutrition, etc).	.115	**.680**	.415	.070	.084
14. I make good decisions about my baby’s health and wellbeing.	**.625**	.116	.102	.035	.074
15. My baby and I have a daily routine	**.458**	.385	.341	.029	−.041
16. I am worried about how others judge me (as a mother).	.026	−.047	.086	−.038	**.798**
17. I can take care of my baby along with my other responsibilities.	.**457**	.123	.452	−.012	−.045
18. Anxiety or worry often disrupts my maternal duties.	.175	.136	−.016	.014	**.780**
19. I become better at taking care of child over time.	.310	**.409**	.165	−.306	−.230
20. I am satisfied with my work as someone who has recently had a baby.	**.767**	.184	.008	.051	.092
**Eigenvalues**	5.25	1.59	1.45	1.21	1.13
**% of variance observed**	18.47	10.73	8.68	8.19	7.11

Factor1: Satisfaction with Maternal Competence, Factor2: Self-care, Factor3: Infant Care, Factor4: Social Support, Factor5: = Maternal Psychological Wellbeing

### Confirmatory factor analysis

The CFA was performed in order to confirm the use of the Persian version of the five-factor BIMF obtained by the EFA in Iranian mothers. The fitness indices of the five-factor model are presented in Table 5. A X^2^/df ratio less than 2 and a RMSEA less than 0.06 confirmed the model validity and a GFI and CFI greater than 0.9, demonstrated the validity of the factor structure and the acceptable fit of the model (Table 4). Therefore, the results of the EFA were supported by the CFA model, and the construct validity of the scale was verified.

**Table 4:** Confirmatory factor analyses fit index of the Persian version of BIMF (*n* = 250)

General fitting indices	x^**2**^/ df	***P***	GFI	CFI	RMSEA
**CFA model**	1.62	< 0/001	0.91	0.91	0.05

*RMSEA* Root mean square error of approximation < 0.06, *GFI* Goodness of Fit Index, and *CFI* comparative fit Index ≥0.90; Chi-square = x^2^, x^2^/ df ≤ 2

**Fig. 2:**
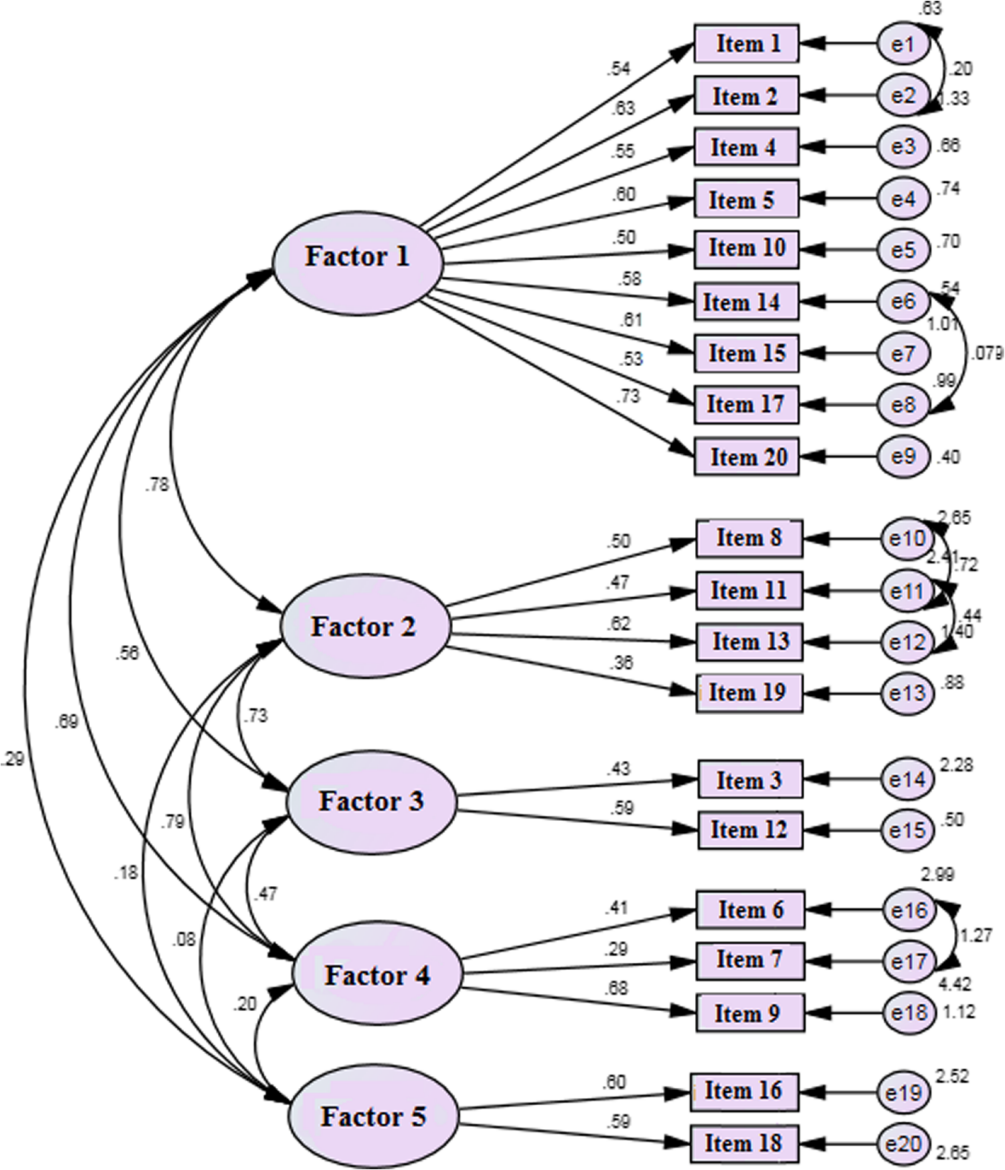
The model of the Persian version of BIMF derived from CFA. *x*^*2*^ *= 1613219; df = 190; p < 0.001; x*^*2*^ */df = 1.61.* Factor 1 = Satisfaction with Maternal Competence. Factor 2 = Self-care. Factor 3 = Infant Care. Factor 4 = Social Support. Factor 5 = Maternal Psychological Wellbeing

Figure 2 illustrates the CFA model for the Persian version of the BIMF with standard coefficients ranging from 0.29 to 0.73 (*p* < 0.001) and variance of the measured errors ranging from 0.20 to 4.42 (*p* < 0.001).

### Reliability

An ICC value of 0.86 (95%CI: 0.77–0.92) and a CR of 0.77 indicated reproducibility, stability and reliability. The total Cronbach’s alpha was 0.80, indicating adequate internal consistency for the Persian version of the BIMF. The Cronbach’s alpha coefficients for the five factors are displayed in Table 5.

**Table 5:** Cronbach’s alpha coefficient and intra-class correlation coefficient for Persian version of BIMF and its subscales

Factors	Cronbach’s alpha	ICC^**a**^(95%CI)	***P***
Satisfaction with Maternal Competence	0.82	0.89(0.83,0.94)	< 0.001
Self-care	0.64	0.79(0.66,0.89)	< 0.001
Infant Care	0.35	0.64(0.38,0.81)	< 0.001
Social Support	0.57	0.78(0.63,0.88)	< 0.001
Maternal Psychological Wellbeing	0.53	0.82(0.69,0.91)	< 0.001
Total Persian version of BIMF	0.80	0.86(0.77,0.92)	< 0.001

^a^Intra-class correlation coefficient

## Discussion

The results of the cultural adaptation and psychometric evaluation of the Barkin Index of Maternal Functioning-Persian Version are reported within this manuscript. Overall, the findings regarding reliability and validity were positive and indicated that the BIMF-Persian version is capable of accurately quantifying the construct of postpartum maternal functioning in Iranian women. These results support the findings of psychometric evaluations (and other validation studies) performed in the United States [2, 22] and Turkey [7]. Currently, the body of evidence indicates that the BIMF has global applicability, though it should be tested in other countries in different regions with different cultures.

A 5-factor structure was obtained as a result of the exploratory factor analysis and included: 1) Satisfaction with Maternal Competence (item 1, 2, 4, 5, 10, 14, 15, 17, 20), 2) Self-care (item 8, 11, 13, 19), 3) Infant Care (item 3, 12), 4) Social Support (item 6, 7, 9), 5) Psychological Wellbeing (item 16, 18). This result is in line with the Aydin et al. study (2018) where a 5-factor solution was also obtained [7]. The agreement between this study and the Aydin et.al. study is intuitive considering the cultural proximity between the two neighboring countries of Iran and Turkey. However, these results are not consistent with the Mirghaforvand et al. and Barkin et al. studies, where 2-factor structure was obtained [4, 41]. The difference between the results of our study and Barkin et al. may be due to large cultural differences between two societies but the different with Mirghaforvand’s study may be related to differences in the study setting and inclusion criteria. Because our study was conducted in Tehran (the capital of Iran) with a multi-cultural texture from all over the country and Semnan in the neighboring province of Tehran. While the study carried out in Tabriz, where the dominant culture of its people is Turkish. Also, this study was conducted on mothers with children aged 2–12 months, while their study was performed on mothers with children aged 6–10 weeks Another distinguishing feature is that our study results did not indicate that the removal of items was necessary. In contrast, Mirghaforvand et al. [41] removed three items (item 15, 16, and 18), and Aydin et al. [7] removed four items (item 15, 16, 18 and 20).

The results of the exploratory factor analysis indicated that the 5-factor model of the Persian version of BIMF accounted for 53.19% of the total variance. Similar value in the Aydin et al. [7], Mirghaforvand et al. [41], and Barkin et al. [4] studies was 59.9, 44.2, and 70.72%, respectively. Therefore, our EFA results were adequate, was acceptable, and in line with similar studies [39].

The Cronbach’s alpha, an indicator of internal consistency, was both adequate and in range with other studies. Studies conducted by Barkin et al. in the United States indicate a Cronbach’s alpha value between .87 and .88 [4, 19]. A study of 530 postpartum Iranian women also reported a Cronbach’s alpha of .88 [41]. In their analysis of 235 Turkish women, Aydin and Kukulu reported a Cronbach’s alpha of .73, which is also in adequate range [39, 40].

The WHO Process of Translation and Adaptation of Instruments [28] and the IQOLA (International Quality of Life Assessment) protocol [29] were used in combination for this project; this method promoted a comprehensive and accurate assessment of the questionnaire items. Expert opinion was obtained and integrated throughout the process and the sample was somewhat diverse in relation to reproductive characteristics; both primiparous and multiparous women were included and both modes of delivery (vaginal and cesarean section) were represented. Including only literate mothers from two urban areas in Iran somewhat limits the generalizability of the findings - although, this measure is performing well in multiple study samples and countries [2, 7, 41].

The strong psychometric properties, ease of administration, and brevity of the BIMF (and, by extension, the Persian version of the BIMF) may implicate this patient-centered measure for widespread use in both medical centers and home visiting programs. Healthcare providers who interact with new mothers such as midwives, obstetrician/gynecologists (OB/GYN) and pediatricians now have a tool at their disposal to evaluate functioning during the postpartum period. While mood disorders such as depression and anxiety should be included as part of routine screening [42, 43], assessment of functioning offers both a different method of evaluation and unique therapeutic option. Providers may decide to address problematic BIMF domains or review the results of all 20 items, once completed. One approach would be to address problematic domains through skill-building exercises. For example, a woman with less than optimal answers on the self-care items might receive targeted, therapeutic support in that specific area.

## Conclusion

Similar to the original English version, the Persian version of the BIMF showed strong psychometric properties. The present analysis adds to the growing body of evidence indicating that the BIMF is a valid and reliable instrument for measuring maternal functioning. The ability to measure functioning, in addition to depressive and anxious symptoms, allows for a more comprehensive assessment of postpartum wellbeing. The relationship between maternal functioning and short- and long-term child health should be examined in future studies.

## Supplementary Information

**Additional file 1.** The Barkin Index of Maternal Functioning-Persian version.

## Data Availability

The data sets generated and analyzed during this study are available from the corresponding author on request.

## Abbreviations

BIMF: Barkin Index of Maternal Functioning
IFSAC: Inventory Functional Status After Childbirth
EFA: Exploratory factor analysis
CFA: Confirmatory factor analysis
CVR: Contentment validity ratio
CVI: Content validity index
ICC: Intra-class correlation coefficient
KMO: Kaiser-Meyer-Olkin
CFI: Comparative fit index
GFI: Goodness of fit index
RMSEA: Root mean square error of approximation
