# Supplementary material for: Persian version of the Barkin Index of Maternal Functioning (BIMF): a cross-cultural adaptation and psychometric evaluation
Source: BMC Pregnancy Childbirth. 2021 Jan 25;21:83. doi: 10.1186/s12884-021-03556-4 (PMC7836187; doi:10.1186/s12884-021-03556-4)
Supplement: Supplementary file 1 — Additional file 1. The Barkin Index of Maternal Functioning-Persian version. [file 12884_2021_3556_MOESM1_ESM.doc]

**The Barkin Index of Maternal Functioning-Persian version**

ID: __________________ Date: ___ / ___ / ______Visit:

Please **circle the number** that best represents how you have felt **over the past two weeks**. Please try to answer each question as honestly as possible as your responses will help us better understand the postpartum experience.

|  | **Strongly Disagree** | **Disagree** | **Somewhat Disagree** | **Neutral** | **Somewhat Agree** | **Agree** | **Strongly Agree** |
| --- | --- | --- | --- | --- | --- | --- | --- |
| 1. I am a good mother. | **0** | 1 | 2 | 3 | 4 | 5 | 6 |
| 2. I feel relieved (rested). | **0** | 1 | 2 | 3 | 4 | 5 | 6 |
| 3. I am satisfied with the way (whether by bottle, through breastfeeding, or both) that I have chosen to feed my baby. | **0** | 1 | 2 | 3 | 4 | 5 | 6 |
| 4. My child and I understand each other. | **0** | 1 | 2 | 3 | 4 | 5 | 6 |
| 5. I can calmly enjoy the time that I spend with my baby. | **0** | 1 | 2 | 3 | 4 | 5 | 6 |
| 6. If I need rest, I can leave my baby’s care without worry to people that I have in my life. | **0** | 1 | 2 | 3 | 4 | 5 | 6 |
| 7. I can comfortably leave my baby’s care to my trusted friends or relatives (This can include the father of the baby or spouse). | **0** | 1 | 2 | 3 | 4 | 5 | 6 |
| 8. I have enough communication with adult | **0** | 1 | 2 | 3 | 4 | 5 | 6 |
| 9. I receive enough encouragement from others | **0** | 1 | 2 | 3 | 4 | 5 | 6 |
| 10. I believe in my inner sense (instincts) while taking care of my child. | **0** | 1 | 2 | 3 | 4 | 5 | 6 |
| 11. In week, I dedicate a time to doing my personal work too. | **0** | 1 | 2 | 3 | 4 | 5 | 6 |
| 12 I can fulfill my baby’s physical needs (such as nutrition, diaper changing, and bringing to the doctor). | **0** | 1 | 2 | 3 | 4 | 5 | 6 |
| 13. I can fulfill my physical needs (such as showering, nutrition, etc). | **0** | 1 | 2 | 3 | 4 | 5 | 6 |
| 14. I make good decisions about my baby’s health  and wellbeing. | **0** | 1 | 2 | 3 | 4 | 5 | 6 |
| 15. My baby and I have a daily routine | **0** | 1 | 2 | 3 | 4 | 5 | 6 |
| 16. I am worried about how others judge me (as a  mother). | **0** | 1 | 2 | 3 | 4 | 5 | 6 |
| 17. I can take care of my baby along with my other  responsibilities. | **0** | 1 | 2 | 3 | 4 | 5 | 6 |
| 18. Anxiety or worry often disrupts my maternal  duties. | **0** | 1 | 2 | 3 | 4 | 5 | 6 |
| 19. I become better at taking care of child over time. | **0** | 1 | 2 | 3 | 4 | 5 | 6 |
| 20. I am satisfied with my work as someone who has recently had a baby. | **0** | 1 | 2 | 3 | 4 | 5 | 6 |
